# Datasets of the environmental factors and management practices of the smallholder tomato production systems in the Colombian Andes

**DOI:** 10.1016/j.dib.2019.103844

**Published:** 2019-03-20

**Authors:** R. Gil, C.R. Bojacá, E. Schrevens

**Affiliations:** a Department de Ciencias Básicas y Modelado, Facultad de Ciencias Naturales e Ingeniería, Universidad de Bogotá Jorge Tadeo Lozano, Bogota, Colombia; b Division of Mechatronics, Biostatistics and Sensors (MeBioS), Faculty of Bioscience Engineering, University of Leuven, Leuven, Belgium

## Abstract

Datasets presented here were employed in the main work “Understanding the heterogeneity of smallholder production systems in the Andean tropics – The case of Colombian tomato growers” Gil, et al., 2019. In this region, tomato crop is developed under two technological levels: low, carried out under open field (*OF*) conditions and, a high, by using greenhouses (*GH*). For *OF*, data belong to five municipalities of the Guanentá province (Santander department), while for *GH*, data belong to five municipalities of the Alto Ricaurte province (Boyacá department). The data presented here includes information on soil parental materials and climate variables (averages ± standard deviations) relevant from the agricultural point of view, which were calculated from historical climate series. Soils natural fertility data, obtained by sampling the production areas, are also presented. After filtering the data, 67 samples were obtained for *OF* and 70 for the *GH*. For *GH*, a dataset with the results of 38 soil samples taken inside greenhouses were paired with the results of samples taken outside these greenhouses in uncropped areas. In the case of these soil analyses, the data correspond to tables with the results reported by the laboratory for both, chemical and physical variables, for each location in which soil samples were taken. In this work, the main dataset is one that contains the inputs of fertilizers and water, and the corresponding yields of tomato production cycles managed by local growers. This information was collected through two data collection tools: surveys (*SVY*) to growers about these aspects in their last production cycle, and through detailed follow-ups of selected production cycles (*FWU*). For the *OF*, we collected data from 71 cycles through the surveys and 22 through the follow-ups, while for the *GH*, information from 138 to 38 tomato cycles was collected through surveys and follow-ups, respectively. A table with the results aggregated by tomato cycle is attached.

**Table:** Specifications table

Subject area	Tropical agriculture, smallholder production
More specific subject area	Soil fertility, crop management, fertilization strategies
Type of data	Tables, figures and text files
How data was acquired	Government agencies, farmer's interviews and on-field measurements
Data format	Raw, filtered and analysed
Experimental factors	We present a table and datasets that describes the environmental heterogeneity in terms of the climate and the natural fertility of the soils for two representative tomato production areas in Colombia. In terms of the soils natural fertility, the experimental factor taken into account was that the samples corresponded to uncultivated soils, seeking to avoid that the results were influenced by the addition of nutrients through fertilization or amendments. We also included data to show the effect of continuous greenhouse tomato production on the soil chemical and physical variables; in this case, the factor corresponds to the place where the samples were taken, i.e. inside or outside the greenhouse. Finally, a tomato production cycles dataset with aggregated inputs and outputs, as well as some cultivation practices is included. This dataset has two factors associated: the production zones and the tool used for data collection, which correspond to surveys and a direct observation procedure called follow-ups.
Experimental features	Soil fertility dataset came from a systematic sampling of the soils in each production region was carried out. Inside and outside soil samples were collected for selected greenhouse tomato production units. Finally, an extensive data collection work was carried out using two data collection tools to learn about the main management practices used by tomato growers, as well as a quantification of the amount of fertilizers used and the yield they are able to obtain.
Data source location	Data from open field tomato were collected in the Guanentá province (latitude: 6º25′18.83″ N, longitude: 73º10′03.18″ W; average altitude: 1370 masl) located in the Santander department, 300 km northwest of Bogotá, Colombia's capital. The main area dedicated to tomato production under greenhouse is located in the department of Boyacá, specifically in the Alto Ricaurte province (latitude: 5º39′37.30″N, longitude: 73º34′57.46″W; average altitude: 2070 masl), located 165 km northwest of Bogotá. There data were collected.
Data accessibility	Data is with this article
Related research article	Gil, R., Bojacá, C.R. and Schrevens, E. 2019. Understanding the heterogeneity of smallholder production systems in the Andean tropics – The case of Colombian tomato growers. NJAS-Wageningen Journal of Life Sciences, https://doi.org/10.1016/j.njas.2019.02.002 [1].

**Table:** 

**Value of the data** • The data serves to quantify the uncertainty derived from soil natural fertility along with the diversity in fertilization practices applied by smallholders. • The data provides the possibility to compare the usefulness of standard data collection tools such as surveys and detailed follow-ups to inventory the inputs and outputs of productions systems lacking a proper record keeping system. • The data allows to compare the efficiency of smallholder production systems, which have different technological intensification level, and at the same time draw a baseline to increase fertilizers use efficiency.

## 1 Data

**Fig. 1:**
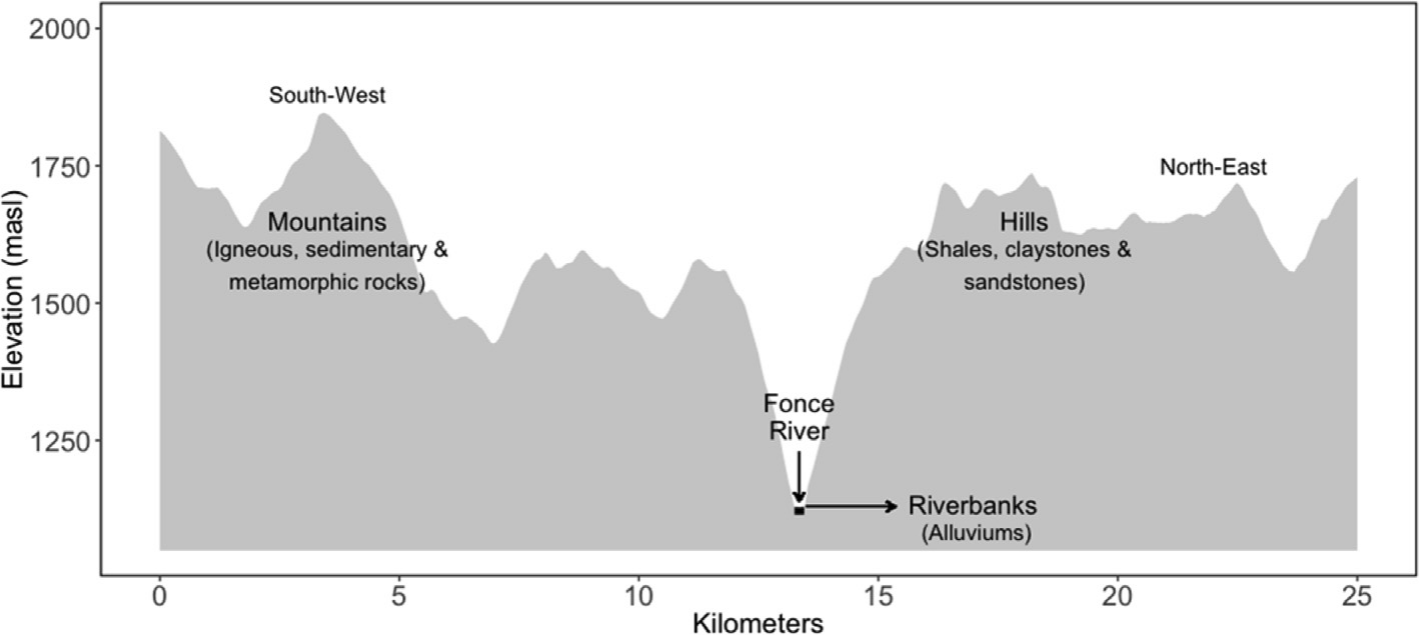
Elevation profile of a cross-section (southwest-northeast direction) of the Guanenta province showing the main types of parent materials.

**Fig. 2:**
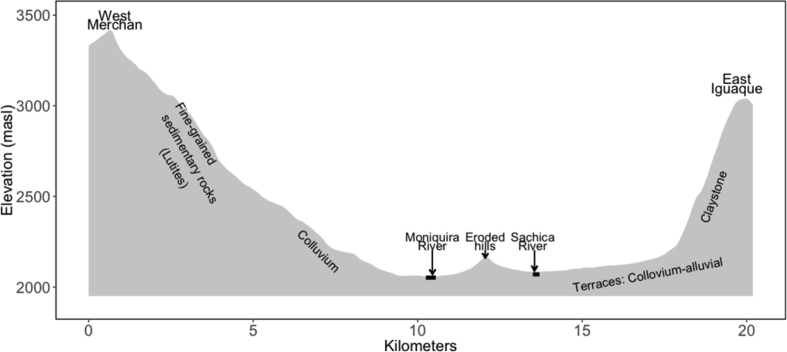
Elevation profile between the massif of Iguaque and the Merchán Mountains, including the main types of parent materials.

The data presented in this article serve to describe the heterogeneity associated with tomato production systems managed by small producers in the Colombian Andes [1]. Data used in this work came from two of the main tomato production areas in Colombia, located in the provinces of Guanentá (Santander department) and Alto Ricaurte (Boyacá department), where tomatoes are cropped under open field (*OF*) and greenhouse (*GH*) conditions, respectively. The distance between the two production zones is 115 km. The landscape of the Guanentá province is formed by mountains and hills crossed by rivers, which in some sectors form small riverbanks (Fig. 1). For *GH* zone, the elevation profile of this study area, including the main soil parent materials, is presented in Fig. 2. In Table 1 the monthly variations of the main agro-climatic variables are presented, from each production area. The datasets attached as supplementary material corresponds to the soil natural fertility defined based on a set of physico-chemical variables (Appendix A) in both production areas (at 30 cm of depth). Also, a table with soil analysis result for lots historically dedicated to tomato production under greenhouse versus nearby uncultivated lots is attached (Appendix B). Finally, we share data related with crop management, fertilizers addition and yields records obtained from surveys and detail follow-ups, recollected on commercial tomato lots (Appendix C).

**Table 1:** Monthly averages (±standard deviations) for the climate variables used to describe the weather in the provinces of Guanentá (Santander) and Alto Ricaurte (Boyacá).

Province	Month	Temperature (ºC)	Relative humidity (%)	Precipitation (mm)	Solar radiation (MJ m^−2^ d^−1^)
Guanentá	Jan	18.7 ± 0.58	78.5 ± 2.59	56.7 ± 5.54	17.9 ± 0.98
	Feb	19.1 ± 0.72	77.5 ± 2.71	92.6 ± 7.82	18.0 ± 1.46
	Mar	19.3 ± 0.66	78.5 ± 2.26	154.8 ± 9.89	17.1 ± 1.19
	Apr	19.2 ± 0.52	80.9 ± 2.10	282.2 ± 13.28	15.8 ± 0.97
	May	19.2 ± 0.50	82.5 ± 2.20	295.1 ± 12.37	15.2 ± 1.15
	Jun	19.0 ± 0.56	82.2 ± 2.15	232.0 ± 10.95	15.2 ± 0.82
	Jul	18.7 ± 0.53	81.7 ± 1.80	223.5 ± 11.08	16.2 ± 0.76
	Aug	18.7 ± 0.56	81.7 ± 2.05	260.3 ± 11.63	16.7 ± 1.07
	Sep	18.7 ± 0.59	81.9 ± 1.93	259.0 ± 11.50	16.6 ± 1.10
	Oct	18.7 ± 0.51	82.6 ± 1.93	304.9 ± 12.52	15.9 ± 0.94
	Nov	18.9 ± 0.53	82.6 ± 1.81	211.8 ± 10.77	15.8 ± 0.86
	Dec	18.7 ± 0.51	81.2 ± 1.67	93.3 ± 7.86	16.8 ± 0.86
Alto Ricaurte	Jan	16.3 ± 0.50	74.0 ± 3.80	46.3 ± 4.72	17.0 ± 1.33
	Feb	16.6 ± 0.61	73.5 ± 4.05	76.9 ± 6.04	17.1 ± 1.45
	Mar	16.7 ± 0.55	75.5 ± 3.36	120.3 ± 7.91	15.9 ± 1.65
	Apr	16.8 ± 0.43	76.8 ± 3.53	150.8 ± 8.71	14.9 ± 1.05
	May	16.8 ± 0.33	77.1 ± 2.94	110.5 ± 7.35	14.6 ± 0.87
	Jun	16.7 ± 0.42	74.0 ± 5.27	53.6 ± 4.89	14.6 ± 0.93
	Jul	16.4 ± 0.29	72.6 ± 5.71	52.0 ± 4.67	15.3 ± 0.96
	Aug	16.5 ± 0.34	71.8 ± 4.41	48.2 ± 4.71	15.8 ± 1.02
	Sep	16.5 ± 0.50	71.9 ± 4.49	78.7 ± 6.32	15.7 ± 1.25
	Oct	16.4 ± 0.38	77.0 ± 4.60	159.2 ± 8.55	14.8 ± 0.97
	Nov	16.3 ± 0.33	78.8 ± 3.23	132.3 ± 7.59	14.4 ± 1.11
	Dec	16.3 ± 0.44	76.7 ± 3.60	86.5 ± 7.11	15.3 ± 1.58

## 2 Experimental design, materials, and methods

### 2.1 Soil and climate data

For each production area, the elevation profiles were constructed with data extracted from Google Earth and supplemented with information about the soil parental materials obtained from previous studies [2], [3], [4]. Climate was described based on historical times series obtained from Colombia meteorological services agency (IDEAM). For the Guanentá province, climate time series were obtained from a weather station placed in Mogotes municipality (6°29′4.79″N, 72°58′28.59″W), while in Alto Ricaurte the station was placed in Villa de Leyva municipality (5°38′37.18″N, 73°34′17.85″W). Time series corresponded to daily records of 50 years for Boyacá (1964–2014) and of 57 years for Santander (1958–2015), but both presented a high proportion of missing data. From the dataset, we excluded years with strong or very strong affection of the El Niño (1957, 1958, 1965, 1966, 1972, 1973, 1982, 1983, 1997, 1998, 2015, 2016) and La Niña (1973, 1974, 1975, 1976, 1988, 1989), based on the Oceanic Niño Index (ONI) calculated and published by the National Oceanic Atmospheric Administration [5]. With the filtered dataset, monthly averages (±standard deviation) were calculated for four variables: precipitation (mm), temperature (ºC), relative humidity (%) and solar radiation (MJ m^−2^ d^−1^). The solar radiation was estimated from the hours of bright sunshine (hours day^−1^) since this variable was not included in the original data. The incoming solar radiation was estimated from extraterrestrial solar radiation and relative sunshine hours, following the equation proposed by Angstrom [6]:

$$I_{t}=I_{et}\left(b_{0}+b_{1}\frac{s}{DL}\right)$$

where $I_{et}$ is the daily total solar radiation above plant canopies (J m^−2^ day^−1^); $I_{et}$ is the extraterrestrial solar radiation (J m^−2^ day^−1^); $b_{0}$ and $b_{1}$ are empirical coefficients, s is the duration of the sunshine (h day^−1^); DL is the day length (hours). The coefficients $b_{0}$ and $b_{1}$ were determined according to climate zones (dry tropical) based in the values proposed by Frere and Popov [7] as 0.25 and 0.45, respectively. For extraterrestrial solar radiation and day length estimation, we followed the equations proposed by Christopher [8], which take into account an eccentricity correction factor and the local solar times.

### 2.2 Soil natural fertility results

Initially, in each production area, 75 soils samples were collected at 30 cm depth on fallow plots between May and July 2015. Sampling spots were determined by a non-aligned random sampling procedure and adjusted once on the field to sample only uncropped soils. Based on the geographic coordinates of the sampling points, we determined the altitude and slope using a 30 m digital elevation model (DEM). Soil samples were processed at a certificated soils laboratory, and the analysis included chemical properties such as nitrate (N—NO_3_), ammonium (N—NH_4_), phosphorus (P) and potassium (K) contents, pH, electrical conductivity (EC), soil organic carbon (SOC); and physical properties such as clay, silt and sand contents (%). Exchangeable N—NH_4_ and N—NO_3_ were determined by extraction with KCl, and the solution was analyzed as described by Bremner and Keeney [9]. Available P was determined by the Bray II method, K by flame photometry, pH and EC were determined in a 1:2 (v/v) soil: water solution, SOC by the wet oxidation method and texture was measured through a hydrometer. P and K contents were transformed to P_2_O_5_ and K_2_O by multiplying them by 2.292 and 1.205, respectively.

Because some samples could correspond to recently cultivated soils and this would cause over-estimates of the natural fertility level, a pre-treatment of the data was carried out to detect and remove outlier records. As we have a multivariate dataset, a method capable of identifying outliers on a matrix composed of n observations and p variables were used. The method used was based on determining the distance in a p-dimensional space taking into account the covariance matrix; specifically, the Mahalanobis distance was calculated using the following formula:

$$MD_{i}=\sqrt{{\left(x_{i}-\mu \right)}^{T}C^{-1}\left(x_{i}-\mu \right)}\;\text{for}\;i=1,\;\text{…,}\;\text{n}$$

where μ is the multivariate arithmetic mean (centroid) and C is the covariance matrix. This distance is used to determine which observations can be considered atypical, as shown by Filzmoser et al. [10] for geochemical multivariable datasets. To define the threshold for atypical observations, we took into account that $MD_{i}^{2}$ approximates to a chi-square distribution with p degrees of freedom [11]. Soil samples having a $MD_{i}^{2}$ greater than $\chi_{p;0.98}^{2}$ were classified as outliers and removed from the dataset. Final datasets comprised 67 and 70 records for Guanentá and Alto Ricaurte provinces, respectively.

### 2.3 Effect of the tomato production under greenhouse on soil properties

As part of the characterization, we determined the effect of *GH* fertilization management on soil properties. We only considered only the *GH* system since tomato is the only crop planted inside these greenhouses throughout the time without any rotation with other crops, and planting can be done at any time throughout the year. Based on the above, changes in soil properties depend exclusively on the fertilization management conducted by the growers. To analyse the effect of *GH* fertilization, we took 30 cm depth soil samples inside *GH* during the fallow period and sampling only *GH* soils with more than two years dedicated to tomato production; and also, on adjacent non-cultivated areas (100–500 m away from the greenhouse edges). The sampling sites were randomly selected based on satellite images on which *GH* locations were clearly identified. Samples were analysed including the same variables used to describe the soil natural fertility in a certified soils laboratory and following the aforementioned methods. We took 38 pairs of soil samples during June 2013.

### 2.4 Fertility management practices

In the present work, two data collection tools were employed: surveys (*SVY*) and a direct follow-up observation procedure (*FWU*). *SVY* consisted on a questionnaire of closed-ended questions about technical aspects related to the last tomato growing cycle such as: cropped area, plant density, cycle length, type and amount of fertilizers applied, crop management practices, water input by irrigation and yield. Questions were redacted by the research team and subsequently were tested through a simulacrum on local growers to improve its comprehension. Once on the field, previously trained undergraduate students conducted the interviews. Between 2009 and 2010, a total of 80 and 174 surveys were carried out to randomly selected smallholder of the *OF* and *GH* technological levels, respectively.

Among surveyed growers, a random subsample was selected for a detailed *FWU* during complete production cycles. The *FWU* method corresponds to a detailed observational procedure on tomato production cycles carried out by members of the research team. *With this data collection tool we* focused on capture data related to use of fertilizers, irrigation, management practices and the obtained yield. At the beginning of each production cycle, we measured characteristics such as cropping area, plant density, irrigation water flow rate, and features associated to available infrastructure. Remaining factors were recorded through weekly interviews with growers, focused on crop management practices including the allocation of time and resources, irrigation schedule, dosing and timing for inputs used in fertilization, as well as fruit production. This procedure was conducted from soil preparation to the end of harvest.

In *OF*, we recorded data for 22 tomato cycles belonging to 10 farms, with four of them being planted consecutively on the same plots. Under *GH* conditions, we recorded data for 39 cycles established in nine farms, with 10 of them being consecutives. The smaller number of consecutive cycles in *OF* is due to the crop rotations scheme applied by each grower. The *FWU* method demanded a higher data collection time than the *SVY* method. *FWU* data collection took 16 months (from October 2011 to February 2013) for the *OF* and 30 months (from September 2010 to March 2013) for the *GH*. From both data collection tools, the data recorded were aggregated in order to obtain the total inputs (e.g. fertilizers) employed for the tomato production along with the total yield achieved. For commercial fertilizers, the nutrient elements contribution was obtained from the official information showed in the label. In the case of organic fertilizers, samples were taken and analyzed to determine the concentration of nitrogen, phosphorus and potassium.

The datasets of soil natural fertility, the effect of the *GH* fertilization strategies on the soils and the data about the management practices in relation to the fertilization are georeferenced. In each table, samples have associated the coordinates in decimal degrees taken in the official geodesic datum for Colombia (MAGNA-SIRGAS).
